# Recent advances in tendinopathy

**DOI:** 10.12703/b/9-16

**Published:** 2020-11-19

**Authors:** Dimitris Challoumas, Mairiosa Biddle, Neal L Millar

**Affiliations:** 1 Institute of Infection, Immunity and Inflammation, College of Medicine, Veterinary and Life Sciences, The University of Glasgow, Glasgow, UK; 2 Department of Orthopaedic Surgery, Queen Elizabeth University Hospital, NHS Greater Glasgow and Clyde, Glasgow, UK

**Keywords:** Tendon, tendinopathy, tendon injury, tendon repair, pathogenesis

## Abstract

Tendinopathy refers to the clinical diagnosis of activity-related pain resulting in a decline in tendon function. In the last few years, much has been published concerning the basic science and clinical investigation of tendinopathy and debates and discussions to new questions and points of view started many years ago. This advances review will discuss the current thinking on the basic science and clinical management of tendinopathy and in particular new findings in the tendon repair space that are relevant to the pathophysiology of tendinopathy. We will further discuss potential novel therapies on the horizon in human tendon disease.

## Introduction

Tendinopathy describes painful conditions that arise in and around tendons in response to overuse and are complex, multifactorial pathologies. Although advances have been made in recent years, it remains challenging to treat, as its definitions, risk factors, and pathophysiology are still evolving. They commonly affect both the upper (shoulder [rotator cuff], elbow, and wrist tendons) and the lower (Achilles, patellar, peroneal, and gluteal tendons) extremities and are associated with a number of different factors, including increasing age, gender, type of exercise and physical activity, occupation, and certain co-morbidities including metabolic or cardiovascular disease^1,2^. The condition is more common in specific sports that involve repetitive loading of a particular tendon or tendon group^1^, and, in the elite sports community, tendinopathy accounts for approximately 30% of the total number of injuries diagnosed^3^. As with specific sports such as volleyball players suffering from knee (patellar) tendinopathy, certain occupations that involve high force with repetitive loading of the tendon, such as manual labourers (joiners/plumbers/bricklayers), musicians, and surgeons, have a higher incidence and prevalence of tendinopathy than the general population^1,4^. This review will provide brief background core information while focusing on updates on the basic science of tendinopathy and tendon repair, in particular findings that may translate to novel therapies on the horizon in human tendon disease.

## Diagnosis

The diagnosis of tendinopathy is largely a clinical one, with the patient often describing activity-provoked localised tendon pain and stiffness. A “typical” tendon history is of pain during the activity, which then often lessens but can be worse the following day, and it can be associated with early morning stiffness. In the early stages, an individual can often continue with the activity, experiencing only intermittent pain. With repeated use of the affected tendon, however, the pain will often progress in nature to a constant debilitating pain and an inability to perform the required activity. For more superficial tendons, palpation is often used as a diagnostic tool, such as thickening in mid portion Achilles tendinopathy. Deeper tendons require pain provocation testing along with additional diagnostic testing to help with determining the diagnosis. Changes that appear on imaging modalities (ultrasound, magnetic resonance imaging [MRI], ultrasound tissue characterisation [UTC], and sonoelastography) do not necessarily correspond to the presence or severity of symptoms^5^. Imaging reveals structural changes and the degree of severity, but pain and aggravating factors need to be considered when clinically interpreting the results. Thus, imaging needs to be placed in the context of the overall clinical picture, and the development of new imaging techniques (UTC or sonoelastography) that utilise more quantifiable parameters will enhance our ability to diagnose, predict the development of symptoms, and monitor the efficacy of tendinopathy treatments.

## Pathophysiology

**Figure 1.**
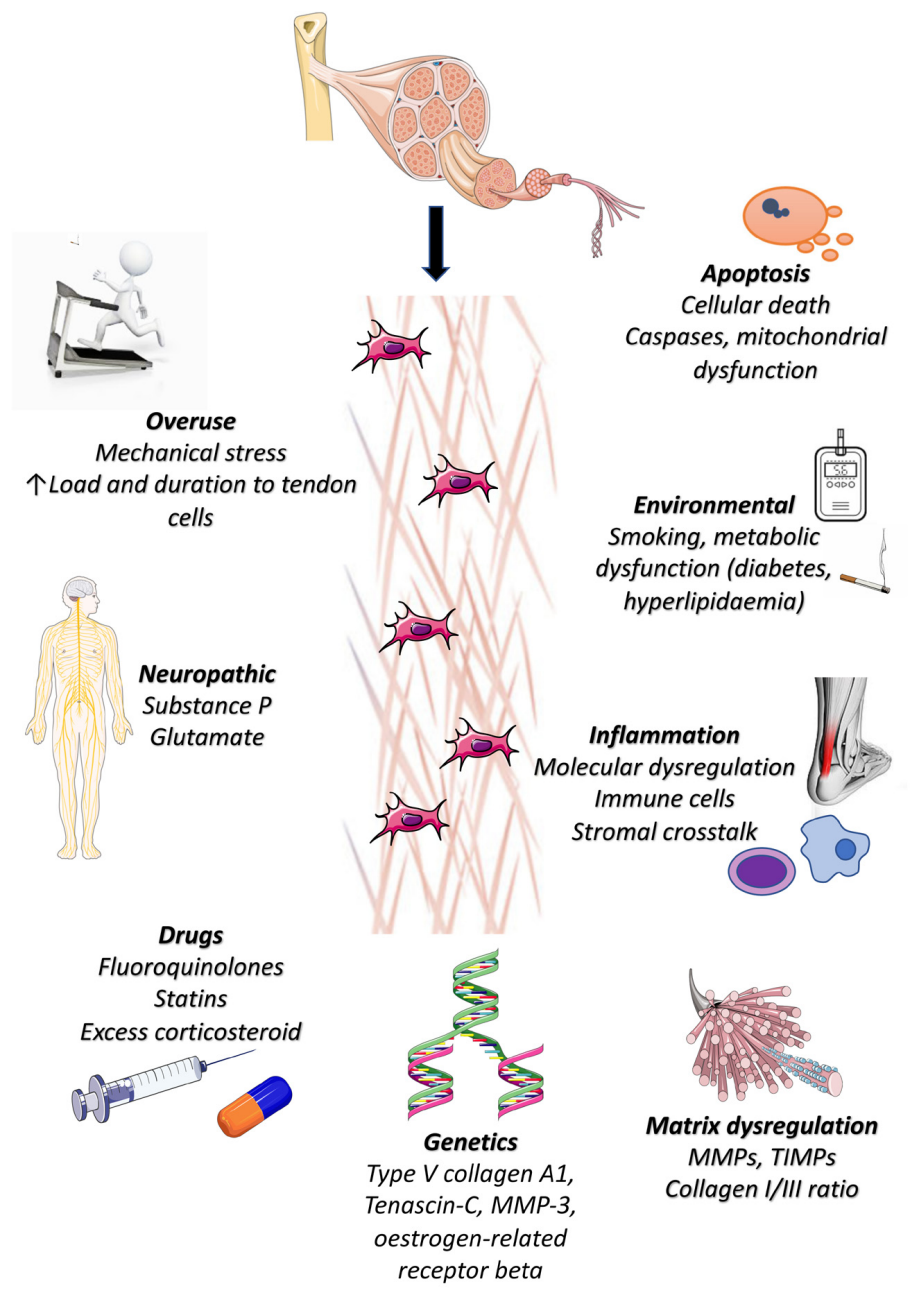
Pathogenesis of tendinopathy. Various risk factors including mechanical overuse, environmental factors including smoking, metabolic disease (diabetes, hyperlipidaemia), and genetics have been implicated in tendinopathy. Additionally, certain medications (fluoroquinolones, statins, excess corticosteroids), neuropathic mediators, excessive apoptosis, dysregulated molecular inflammation, and matrix production are all implicated in the development of tendinopathy. MMP, matrix metalloproteinase; TIMP, tissue inhibitors of metalloproteinases.

The pathogenesis of tendinopathy appears to be multifactorial (Figure 1), and despite the growing body of research aiming to elucidate its exact aetiology and underlying mechanisms, several questions remain unanswered.

Various theories have been proposed with regard to the pathogenesis of tendinopathy. The vast majority of them fall into one of three models based on the primary event in the pathology cascade: a) collagen disruption/tearing model, b) inflammation, and c) tendon cell response. The “failed healing” theory proposed by Fu *et al*. is a unified theory incorporating various previously proposed ones^6^. It suggests that it all starts with an initial injury (or repetitive microinjuries) and an unfavourable mechanical environment. The normal healing process is diverted into an abnormal pathway due to unfavourable mechanical environment, disturbances of local inflammatory responses, oxidative stress, and pharmacological influences. This abnormal healing then leads to pathological changes in the tendon matrix, cytokine profiles, vascularity, innervation, cellularity, and cell phenotypes, which in turn lead to clinical presentation, which is either pain (a result of increased nociception) or rupture (due to mechanical weakness)^6^. Similarly, the “continuum model” of tendon pathology proposed by Cook *et al*. is also a popular unified theory^7,8^. According to this, there are three stages to tendon pathology with continuity between them: a) reactive tendinopathy, b) tendon dysrepair (failed healing), and c) degenerative tendinopathy. In the first stage, there is a non-inflammatory proliferative response in the cell and matrix that occurs with acute overload and leads to adaptive thickening of a portion of the tendon. In the second stage, there is matrix breakdown, an increase in the number of cells, and production of proteoglycans and collagen, which is now disorganised. There may also be neoangiogenesis and neuronal ingrowth. In the last stage, areas of cell death occur because of apoptosis and tenocyte exhaustion, and alongside the increasing disorganisation of the matrix this results in matrix heterogeneity with little capacity for reversibility of the damage^7,8^. This latter model does not, however, recognise the role of inflammation in tendinopathy, which has been demonstrated in numerous studies^9^.

Regardless of the exact underlying model, animal and human studies have identified several factors at a cellular and molecular level that appear to contribute to or are associated with the development of tendinopathy. Histological analyses of diseased tendon samples have demonstrated disorganised collagen bundles, collagen fibre fragmentation, replacement of type I with type III collagen, neovascularity, and neoinnervation^10,11^. Relevant research has focused on identifying transcription factors and signalling pathways regulating the formation of type I collagen and its architecture within the extracellular matrix (ECM) of the tendon, and some transcription factors implicated in the modulation of *COL1A1/A2* gene expression have been identified (e.g. scleraxis and Mohawk homeobox protein)^12,13^. The non-collagenous ECM (elastin, glycoproteins, proteoglycans, etc.) is also thought to be implicated, as its components have been found to play a role in both ECM assembly and the regulation of cell growth and differentiation^14^.

Oxidative injury and mitochondrial dysfunction are also implicated. Degenerative tendon produces reactive oxygen species and oxygen free radicals, which in turn can lead to stress-induced apoptotic cell pathways^15^. Apoptosis is thought to cause a progressive loss of intrinsic tendon cells and, indeed, degenerative rotator cuff tendon samples have been shown to demonstrate increased expression of caspase 3 and 8, which are known to be initiators of apoptosis^16,17^. Mitochondrial membrane potential has been shown to be decreased in human samples of degenerative tendon, and the expression of BNIP3 (BCL2-interacting protein 3), which is implicated in pro-apoptotic pathways and mitochondrial dysfunction, has been found to be upregulated^18^. Finally, nitric oxide (NO), which is involved in inflammation, is produced by NO synthase (NOS), the activity of which is increased in tendinopathy^19^. This has been repeatedly demonstrated in both diseased human tissue and animal models, and, additionally, NO exposure has been shown to increase the production of various tendon ECM components^20^.

Signalling pathways have also been implicated in the pathogenesis of tendinopathy. Wnt signalling has been reported to play a role with overexpression of Wnt3a and beta-catenin in patellar tendinopathy samples and so has the NF-kB pathway in human shoulder tendinopathy samples and mouse tendon cells^21,22^. Finally, rodent tendinopathy models demonstrated overexpression of ERK1/2 signalling and inhibition of p38; the former is thought to have an important role in the action of steroids inhibiting proliferation and collagen synthesis in tenocytes and the latter has an effect on the expression of ECM and cell proliferation genes^23,24^. Additionally, genetic predisposition is thought to be implicated; a number of genetic polymorphisms (*COL5A1*, *MMP-3*, *TIMP2*, and tenascin-C) that disturb tendon homeostasis and its healing ability following mechanical overload and injury have been identified^25–27^.

Molecular inflammation and the immune system have been shown to play a fundamental role in tendinopathy. Both animal models and clinical samples of diseased human tendons have shown that tendinopathy is not the result of mere mechanical degeneration but involves complex immunological interactions with the influx of innate and adaptive immune cells being shown to play important roles^9,28^. These are thought to convert the initial healthy healing response of diseased tendon into chronic symptomatic disease by the production of various inflammatory cytokines and chemokines, which alter the microstructure of tendon. Macrophages and mast cells (both local and systemic) are immune cell types that have been shown to result in overproduction of inflammatory cells within the tendon and stimulate a chronic inflammatory response^29^. A plethora of interleukins and other cytokines are involved in the pathogenesis of tendinopathy, some of which are IL-1beta, IL-4, IL-6, and TNF-alpha^30^. IL-17 is thought to be of paramount importance, as it may play a role in ECM remodelling through type III collagen production and initiation of other pro-inflammatory cytokines^31^. Anti-IL-17 agents are currently being investigated for the treatment of tendinopathy^32^. Finally, alarmins (damage-associated proteins), which are known to be released rapidly after non-programmed cell death (necrosis), are key initiators of the innate immune system, and restore tissue homeostasis, have also been implicated^33^.

The underlying mechanism of perceived pain as a result of tendinopathy is also incompletely understood. The presence and severity of pain do not always correlate with the extent of the underlying structural changes, and, indeed, structural changes are not a prerequisite for the development of pain. In addition, progression of tendon pathology over time is much more likely to lead to symptomatic tendinopathy than the absolute extent of pathology at a single time point^8^. Upregulation of glutamate and its receptors has been found in diseased tendon along with other nociceptive mediators such as metabotropic glutamate receptor 2, kainite receptor 1, protein gene 9.5, and substance P, and they are thought to be implicated in the underlying pathologic changes in addition to mediating pain^34^.

## Management

Current management can be divided into active rehabilitation, which mainly involves tendon-loading physiotherapy regimes along with patient education and intervention strategies, including steroid injection, medications, platelet-rich plasma, iontophoresis, extracorporeal shockwave therapy (ESWT), low-energy laser therapy, and therapeutic ultrasound. Importantly, the treating clinician should identify any precipitating factors such as sudden acute changes in load (change in training regimes), previous injury, medication changes (increased tendinopathy risk related to the use of fluoroquinolones, antibiotics, or steroids), family history of inflammatory arthritis, and any relevant medical history (increased tendinopathy risk associated with diabetes, smoking, hyperlipidaemia, and obesity).

### Exercise-based strategies and other non-surgical modalities

A tailored, individualised exercise regime based on current evidence-based principles of load and exercise progression is most likely to ensure patient compliance and therefore benefit. Eccentric tendon-loading exercise regimes remain one of the most effective conservative therapies for tendinopathy. Results have shown benefit when applied to Achilles and patellar tendinopathies of the lower limb^35,36^, along with lateral elbow and shoulder tendinopathy in the upper limb^37,38^. The addition of isometric exercises into a rehabilitation programme has been shown to be of benefit, in particular with athletes suffering from patellar tendinopathy^39,40^. These positive findings, however, have not been reproduced in more recent studies and therefore its role remains ambiguous^41^. Another strategy, heavy slow resistance training (HSRT), allows the tendon to be subjected to greater volumes of loading with fewer repetitions. This results in a greater time under tension, leading to greater tendon adaptation, and can be considered in a progressive loading regime^42^.

Corticosteroids are often used to provide short-term pain relief, allowing the patient to begin an exercise regime; however, its role is still debated. This is because of certain studies showing no benefit of steroid injection versus control^43^ with regard to pain and return to normal tasks, along with the detrimental effects of the steroid on tendon function^44^ and the potential of tendon rupture^45^.

Topical glyceryl trinitrate (GTN) is considered a safe and reliable treatment or adjunct for the management of tendinopathy. A recent systematic review of randomised controlled trials (RCTs) showed significant improvements in pain when comparing GTN with placebo in the short term, along with further significant improvements for up to 6 months. It can, however, be associated with an increased incidence of headaches^46^.

Platelet-rich plasma is thought to promote tendon healing; however, to date, there is no high-quality evidence to support its use^47^. Iontophoresis is often suggested for more superficial tendons, and its aim is to induce pain relief. Again, there is insufficient evidence to support its use as a stand-alone treatment, but its low side effect profile lends itself to being used as an adjunct therapy^48^.

ESWT has been used as a treatment for tendinopathy and soft tissue disorders of the shoulder, elbow, hip, knee, and ankle areas. The exact mechanism of ESWT is not fully understood; however, one study suggests that the mechanical stimulus provided by ESWT might aid tendon remodelling in tendinopathy by promoting the inflammatory and catabolic processes that are associated with removing damaged matrix constituents^49^. It appears to have increased efficacy when used more frequently and at an increased dose. It also appears to show increased benefit in treating calcific tendinopathy compared with non-calcific tendinopathy^47^. Currently, its use is considered safe as an adjunct, but again further high-quality studies are required to determine the optimum treatment protocol.

Low-level laser therapy (LLLT) uses light energy at levels low enough to not cause skin temperature increases, and there is some evidence for its ability to reduce inflammation and oedema, induce analgesia, and promote healing in a range of musculoskeletal pathologies. A small systematic review concluded that LLLT compared to placebo was an effective treatment option for tendinopathy^50^. There have also been promising results using low-intensity pulsed ultrasound in the treatment of Achilles tendinopathy^51^, but once again further research needs to be carried out to determine the long-term benefits and disadvantages of these alternative therapies. High-volume injection (HVI) is a treatment involving the injection of a large volume of saline, usually mixed with corticosteroid and/or local anaesthetic; however, nearly all studies on HVI consist of relatively small numbers of patients, limiting the generalizability of this treatment modality.

### Surgery

The aim of surgery is to promote regeneration of the tendon. Procedures vary from open procedures to minimally invasive operations through percutaneous incisions or with arthroscopy, depending on the site of tendinopathy.

No RCTs are available comparing surgery with non-operative management for Achilles tendinopathy. Surgery for this tendinopathy often involves tenotomy and debridement of the diseased portion. There has been no benefit demonstrated in radiofrequency microdebridement of this tendon in comparison to surgical decompression^52^. Patellar tendinopathy is also most commonly treated with tenotomy and debridement. A recent Cochrane review, however, demonstrated no benefit of open surgery versus eccentric exercises for patellar tendinopathy at 12 months with regard to pain and tendon function^53^.

Similarly, in the upper limb, lateral elbow tendinopathy is treated with debridement and excision of extensor carpi radialis brevis. However, a prospective, randomised, double-blinded, placebo-controlled clinical trial found no benefit of the excision surgery in comparison to the placebo surgery^54^. The CSAW trial for shoulder tendinopathy also demonstrated that although surgical groups had better outcomes for shoulder pain and function, it was not statistically significant. Surgical decompression also showed no increased benefit over arthroscopy only, questioning the value of the operation for the condition^55^. Finally, a recent systematic review of RCTs showed that surgery does not appear to be superior to physiotherapy in the mid or long term, and loading therapy (with or without adjuncts) is recommended for at least 12 months before surgery is considered^56^.

## Strategies for the management of tendinopathy and future direction

The future of tendinopathy research requires that the findings from basic laboratory research (both acute tendon injury and chronic disease models) be integrated into the clinical research domain to provide a comprehensive picture of the disease process involved in tendinopathy. Thus, results from genetic, epigenetic, *in vitro*/*vivo*, epidemiological, observational, and clinical studies need integration into logical pathways to treat the disease.

There has been increasing attention on tendon healing strategies recently directed towards both tendinopathy and tendon ruptures. Biologic therapies include the use of scaffolds and delivery of genes, growth factors, and cells. Scaffolds can be used to deliver mechanical support by providing a suitable environment for the attachment, proliferation, and subsequent migration of cells to establish a base for matrix remodelling and ultimately native tendon tissue regeneration. In addition to the lack of long-term follow up, findings in clinical studies using scaffolds have been inconsistent with respect to clinical outcomes and adverse effects^57^. Approximately one-quarter of patients who received non-cross-linked porcine scaffolds experienced significant aseptic inflammatory reactions; therefore, their use is not recommended^57,58^. Various materials are available, and, although natural ECM scaffolds were initially favoured, synthetic scaffolds seem to be very promising^59,60^.

Gene therapy remains an experimental treatment approach with no licenced clinical application as yet. It can be performed through direct gene transfer or viral and non-viral vectors coding for growth factors, usually administered with the means of local injections. They are designed to supply exogenous genetic materials into cells in order to subsequently alter the DNA and induce, silence, upregulate, or downregulate the expression profile and secretion of proteins. Several factors have been tested in animal studies and yielded promising results, including, but not limited to, BMP12, bone marrow-derived mesenchymal stem cells, and TGF-beta1^61–63^. Growth factors have the capacity to promote the differentiation of local stem cells into fibroblasts and therefore accelerate tendon healing.

Finally, the use of stem cells is an interesting strategy for tendon repair and regeneration, and studies have reported promising results with regard to their effects and safety^64,65^. There are two different types of mesenchymal stem cells that are commonly used: bone marrow- and adipose tissue-derived cells. The exact mechanism of action of stem cells remains unknown; therefore, more basic science is needed before they are used in meaningful clinical trials. The therapeutic effects of stem cells in target tissue are thought to be exerted at least in part through paracrine actions, such as through exosomes, which are extracellular vesicles^66^. Exosomes mediate intercellular communication by delivering functional molecules such as proteins and mRNAs to recipient cells, and they are thought to be both effective and safe^67,68^.

All the aforementioned novel healing strategies are promising treatments for tendinopathy. More detailed understanding of the underlying pathogenesis and basic science of tendinopathy will guide the development of clinically effective cellular and molecular treatment strategies that will restore injured tendons to their premorbid state. Further delineation of associated mutations and epigenetic changes could also facilitate future research to identify effective therapies and DNA biomarkers for earlier diagnosis. Exosomes may be used as delivery vehicles for tendon-targeted gene therapy in environments suitable for best possible effects. Furthermore, the ideal stem cell type, cell number, and scaffold material are areas of ongoing investigation and have significant potential. The majority of these biologic strategies have been applied to models of acute disease, especially the repair of tendon ruptures, and perhaps more attention should be paid to chronic, degenerative tendinopathy, which is much more common in clinical practice. Finally, the use of disease-modifying agents such as chondrolytics for targeted removal of mucoid deposits may be investigated in the future, along with the application of more than one mode of biologic therapy simultaneously capitalising on potential synergistic effects^57^.

## Conclusion

Despite the increasing attention of the research community towards understanding and treating tendinopathy, several questions remain unanswered. It constitutes a complex condition with regard to its pathogenesis and pathophysiology, which makes it so challenging to treat. Whilst loading regimes will remain the mainstay of clinical treatment, new biologic and tissue-engineering therapies are constantly emerging, some of which have shown promise in pre-clinical and clinical settings, which will provide important adjuncts for those 30–50% of patients who may ultimately fail loading regimes. More detailed understanding of the underlying basic science through laboratory-based research will undoubtedly be of paramount importance in guiding the identification and application of effective therapies.
